# Metagenomic next-generation sequencing to detect *Pneumocystis jirovecii* pneumonia in critically ill, HIV-negative children: a retrospective multicenter study

**DOI:** 10.1186/s12890-026-04163-9

**Published:** 2026-02-07

**Authors:** Liming He, Yibing Cheng, Li Huang, Zhenyu Zhang, Qunqun Zhang, Ling Gong, Tian Li, Xiulan Lu, Xiaodi Cai, Gangfeng Yan

**Affiliations:** 1 Department of Pediatric Intensive Care Unit, Children’s Hospital of Fudan University, Shanghai, China; 2 Department of Pediatric Intensive Care Unit, Henan Children’s Hospital, Zhengzhou, China; 3 Department of Pediatric Intensive Care Unit, Guangzhou Women and Children’s medical center, Guangzhou Medical University, Guangzhou, China; 4 Department of Pediatric Intensive Care Unit, Hunan Children’s Hospital, Changsha, China

**Keywords:** Metagenomic next-generation sequencing, *Pneumocystis jirovecii* pneumonia, Non-human immunodeficiency virus, Children

## Abstract

**Background:**

Metagenomic next-generation sequencing (mNGS) plays a critical role in the rapid detection of infectious pathogens. We aimed to analyze the clinical characteristics of *Pneumocystis jirovecii* infection in children without HIV infection and to evaluate the performance of mNGS in distinguishing *P. jirovecii* colonization from true infection.

**Methods:**

A multicenter, retrospective analysis was conducted on critically ill, non-HIV-infected pediatric patients who tested positive for *P. jirovecii* via mNGS analysis of bronchoalveolar lavage fluid (BALF). Group differences were assessed using Mann–Whitney U-tests (for continuous data) and chi-square tests (for categorical data). Discriminatory performance was evaluated by calculating the area under the receiver operating characteristic curve.

**Results:**

A total of 59 HIV-negative children (age range: 2 months to 14 years) from four children’s hospitals were included and classified into two groups based on *P. jirovecii* status: *P. jirovecii* pneumonia (PCP; *n* = 51) and *P. jirovecii* colonization (PCC; *n* = 8). Compared with the PCC group, the PCP group had significantly higher serum C-reactive protein levels and median *P. jirovecii* read counts in mNGS (both *P* < 0.05). The optimal threshold value for discriminating *P. jirovecii* infection from colonization appeared to be 556 reads (sensitivity, 77.6%; specificity, 100.0%). Eighteen patients (35.3%) in the PCP group died. Compared with survivors, these patients were significantly younger, had lower T-cell subset counts (CD3^+^, CD4^+^, and CD8^+^), and a higher prevalence of primary immunodeficiency (all *P* < 0.05).

**Conclusions:**

BALF mNGS analysis may have utility for differentiating between colonization and infection by *P. jirovecii*, warranting further investigation.

**Supplementary Information:**

The online version contains supplementary material available at 10.1186/s12890-026-04163-9.

## Introduction

*Pneumocystis jirovecii* is an opportunistic pathogen that can cause *P. jirovecii* pneumonia (PCP) in immunocompromised patients [1]. Since the introduction of highly active antiretroviral therapy, PCP incidence has declined among people living with HIV. In contrast, the widespread use of immunosuppressants and chemotherapeutics in patients with solid tumors, hematologic malignancies, rheumatic diseases, and other conditions has contributed to a rising incidence of PCP among immunocompromised individuals without HIV infection [2, 3]. Non-HIV-infected patients with PCP typically develop a more acute disease course and have a higher mortality rate (28%–53%) than those living with HIV (17%–30%) [4–6]. The main symptoms of PCP include fever, dry cough, chest pain, and dyspnea. Radiologically, the most common findings are bilateral, diffuse ground-glass opacity, while small nodules, unilateral infiltrates, and consolidations are less frequently observed [6].

The prognosis of PCP in HIV-negative patients depends on timely diagnosis and treatment, appropriate prophylaxis for high-risk populations, and early identification of severe cases [2]. *P. jirovecii* mainly parasitizes the pulmonary alveoli in the form of cysts and trophozoites. Microscopic visualization of cysts and trophozoites in respiratory specimens, such as bronchoalveolar lavage fluid (BALF) or sputum, has long been considered the gold standard for PCP diagnosis; however, its low sensitivity results in a limited positive detection rate [7]. With the rapid development of metagenomic next-generation sequencing (mNGS) technology to detect target organisms in critically ill patients, research on the application of mNGS in *P. jirovecii* detection has emerged [8, 9]. However, to our knowledge, the few reports on the performance of mNGS for PCP diagnosis using BALF in critically ill, non-HIV-infected pediatric patients all described small sample, single-center studies [10–12]. Furthermore, the use of mNGS to distinguish *P. jirovecii* colonization from true infection remains challenging. An adult study evaluating the clinical performance of mNGS and serum 1,3-β-D-glucan (BDG) for discriminating *P. jirovecii* infection from colonization showed that mNGS could discriminate infection from colonization using a cut-off of 14 reads [13].

Because no relevant studies have addressed pediatric populations, we conducted a retrospective analysis of HIV-negative children admitted to the pediatric intensive care units (PICUs) of four children’s hospitals between January 2019 and May 2023, all of whom tested positive for *P. jirovecii* in BALF via mNGS. We aimed to characterize the clinical features of PCP and to evaluate the performance of mNGS in distinguishing *P. jirovecii* infection from colonization in this critically ill cohort.

## Materials and methods

### Study population

This multicenter, retrospective study was conducted in the PICUs of Children’s Hospital of Fudan University, Guangzhou Women and Children’s Medical Center, Henan Children’s Hospital, and Hunan Children’s Hospital from January 2019 to May 2023. Patients with severe pneumonia who tested positive for *P. jirovecii* via mNGS of BALF were enrolled. Bronchoscopy with BALF collection followed by mNGS was performed in critically ill children with suspected severe pneumonia, defined by the need for noninvasive/invasive ventilatory support, radiographic evidence of significant pulmonary infiltration, and unidentified etiology.

### Inclusion and exclusion criteria

Based on the revised European Organisation for Research and Treatment of Cancer/Mycoses Study Group Education and Research Consortium (EORTC/MSGERC) consensus definitions of PCP in individuals without HIV, the patients were divided into probable PCP and *P. jirovecii* colonization (PCC) groups [6]. Inclusion in the PCP group was defined by the following criteria: (1) immunosuppression without HIV infection; (2) presence of cough, fever, and shortness of breath; (3) radiologic findings of diffuse ground-glass opacity with interstitial infiltrates, along with consolidated or reticulate shadows in both lungs; (4) and two positive serum BDG tests with levels ≥ 80 pg/mL. Patients were assigned to the PCC group if *P. jirovecii* was detected via mNGS of BALF in the absence of appropriate host factors or supporting clinical and radiologic features. Exclusion criteria were as follows: (1) HIV infection; (2) mNGS sample not derived from BALF; (3) no serum BDG assay results; and (4) incomplete medical record. The diagnosis of PCP or PCC was made by a multidisciplinary panel comprising two senior pulmonologists and one senior infectious disease specialist after reviewing all clinical, radiological, laboratory and microbiological data.

Immunosuppression was considered when at least one of the following criteria was met: (1) CD4 count < 200 cells/µL; (2) hematologic malignancy; (3) primary immunodeficiency; (4) post- transplantation status; (5) history of immunosuppressive agent treatment; (6) daily dosage of ≥ 0.3 mg/kg prednisone equivalent for ≥ 14 days; and (7) neutropenia [12]. Primary immunodeficiency was defined by the presence of a pathogenic gene, as per the 2015 classification criteria for primary immunodeficiency diseases published by the International Union of Immunological Societies [14].

### BALF collection and preservation

BALF was collected by experienced respiratory therapists following established guidelines [15, 16]. Quality requirements for BALF sampling were as follows: (1) a ≥ 40% recovery rate; (2) absence of blood mixing (< 10% red blood cells); (3) collection from diseased lung segments (based on recent chest computed tomography [CT] results) or from the right middle lobe for diffuse lung disease; (4) lavage volume of 1 mL/kg (≤ 20 mL per aliquot), with a total instilled volume of 5–10 mL/kg; and (5) collection into a sterile container for analysis within 2 h. Specimens that could not be sent within 2 h could be stored in a 4 °C refrigerator and analyzed within 24 h.

### mNGS analysis

We performed mNGS following our previously published protocol [17, 18]. Briefly, a BALF aliquot (0.6–3 mL) was inactivated and mixed with glass grinding beads to break microbial cell walls. DNA was then extracted using the TIANamp Micro DNA Kit (Tiangen Biochemical Technology, Beijing, China), in accordance with the kit instructions. To construct libraries, the end repair, ligation, and PCR amplification of DNA fragments were performed using the MGIEasy Cell-free DNA Library Prep Kit (MGI Tech Co., Ltd., Shenzhen, China), in accordance with the kit instructions. Libraries were quality controlled using the 2100 Bioanalyzer (Agilent, Santa Clara, CA, USA) to confirm a fragment size of 200–300 bp, and quantified using the Qubit dsDNA HS Assay Kit (Thermo Fisher Scientific, Waltham, MA, USA) to ensure a DNA library concentration > 2 ng/µL. Equal amounts of nucleic acid from each sample were applied for subsequent operations. High-throughput sequencing was performed on a BGISEQ-50 (MGI Tech Co., Ltd.), following the manufacturer’s instructions. Sequencing data pre-processing included quality control to remove low-quality and short reads (< 35 bp), yielding high-quality sequences. These reads were then aligned to the human reference genome (hg19) using BWA software (version 0.7.15-r1140) to eliminate human-derived contamination. After removing repetitive and low-complexity sequences using PRINSEQ software (version 0.20.4), the remaining reads were aligned to the PMDB pathogen database, an internal reference of the Beijing Genomics Institute containing 4,945 viruses, 6,039 bacteria, 1,064 fungal species, 234 protozoal species, 174 *Mycobacterium* species, and 137 *Mycoplasma* species. Microbial sequence data obtained after alignment were taxonomically classified into viruses, bacteria, fungi, and parasites.

### Clinical data collection

Within 1 week of obtaining mNGS results, we collected patient data including demographics, underlying conditions, clinical symptoms and signs, laboratory test results, and radiologic findings. Additional data included Pediatric Logistic Organ Dysfunction-2 (PELOD-2) scores for inpatients, prior use of glucocorticoids or immunosuppressants, time from symptom onset to initiation of trimethoprim and sulfamethoxazole (TMP/SMZ), results of pathogen testing, and outcomes at discharge.

### Statistical analysis

Analyses were carried out using the SPSS platform version 23.0 (SPSS, Inc., Chicago, IL, USA). Normally distributed variables were compared using a Student’s *t*-test and are expressed as mean ± standard deviation. Nonparametric continuous variables were compared using a Mann–Whitney U-test and are presented as median (interquartile range [IQR]) values. Categorical variables were compared using a χ^2^ test or Fisher’s exact probability test and are expressed as percentages. Multivariate logistic regression was employed to analyze mortality factors. Receiver operating characteristic curve (ROC) analysis was used to assess the value of BALF mNGS in differentiating between *P. jirovecii* infection and colonization, and the Youden index was applied to determine the optimal read-count threshold along with its corresponding sensitivity and specificity. A *P* value < 0.05 was considered statistically significant.

## Results

### Population characteristics

**Fig. 1:**
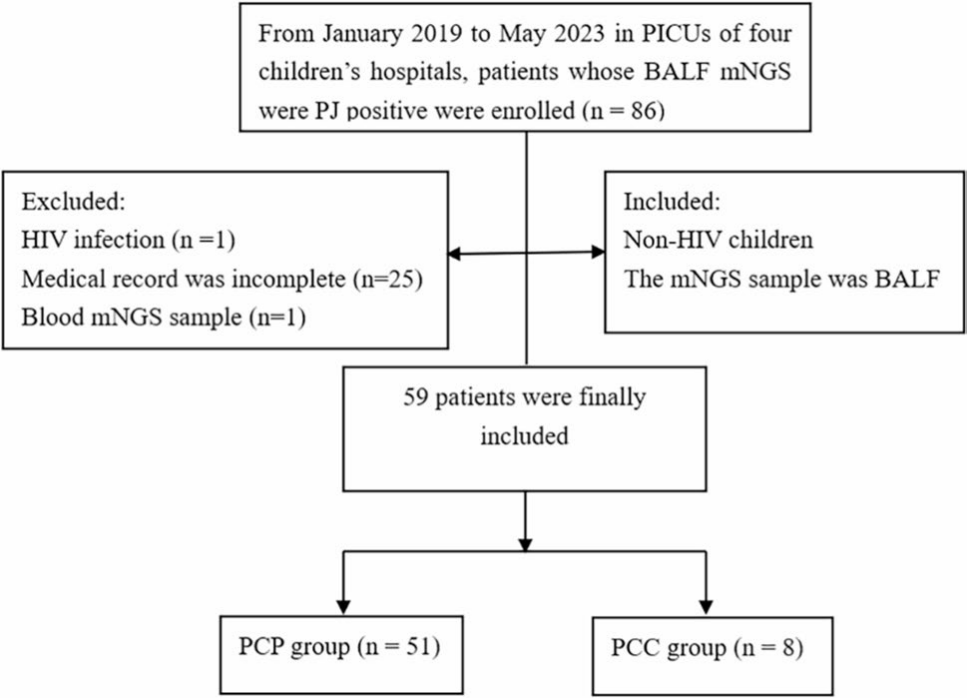
Flow chart of patient selection for the study. PICU: pediatric intensive care unit; BALF: bronchoalveolar lavage fluid; mNGS: metagenomic next-generation sequencing; PJ: *Pneumocystis jirovecii*; HIV: human immunodeficiency virus; PCP: *P. jirovecii* pneumonia; PCC: *P. jirovecii* colonization

From January 2019 to May 2023, a total of 686 patients underwent BALF mNGS testing, of whom 86 (12.5%) patients tested positive for *P. jirovecii*. Among these, one patient was infected with HIV, one underwent mNGS analysis of a blood sample, and 25 patients lacked original reports, resulting in a final enrollment of 59 pediatric patients (Fig. 1). Based on the 2021 EORTC/MSGERC definition of PCP, the patients were assigned to the PCP (*n* = 51) and PCC (*n* = 8) groups. Forty-two patients were from Children’s Hospital of Fudan University (40 in the PCP group and two in the PCC group), five from Guangzhou Women and Children’s Medical Center (four in PCP and one in PCC), five from Hunan Children’s Hospital (four in PCP group and one in PCC), and seven from Henan Children’s Hospital (three in PCP and four in PCC). Patient age ranged from 2 months to 14 years (median: 10 months), and most (42/59, 71.2%) were males. Underlying conditions included primary immunodeficiency disease (*n* = 24), solid tumors (*n* = 14), hematologic malignancies (*n* = 4), hematopoietic stem cell transplantation (HSCT) (*n* = 2), solid organ transplantation (*n* = 3), rheumatic disease (*n* = 2), nephrotic syndrome (*n* = 2), and inherited metabolic disease (*n* = 2). Fifty-two cases (88.1%) involved fever. Invasive/noninvasive mechanical ventilation was required in 51 cases (86.4%). Coinfections were detected via mNGS of BALF in 46/59 (78.0%) cases, involving the following top five pathogens: cytomegalovirus (*n* = 21, 35.6%), Epstein–Barr virus (*n* = 6, 10.2%), *Acinetobacter baumannii* (*n* = 5, 8.5%), *Mycobacterium tuberculosis* (*n* = 4, 6.8%), and *Streptococcus pneumoniae* (*n* = 4, 6.8%). The antimicrobial regimen was adjusted for 26 patients (44.1%) based on mNGS results combined with clinical and radiologic manifestations (Table 1). Supplementary Table 1 showed the distribution of specific co-detected pathogens, stratified by the PCP and PCC groups.

**Table 1:** Demographic and clinical characteristics of the study patients

Variables	Total
Cases	59(PCP:51, PCC:8)
Male, n(%)	42(71.2)
Age, months	10(5, 47)
Number of people included in each hospital, n	
Children’s Hospital of Fudan University	42(PCP:40, PCC:2)
Henan Children’s Hospita	7(PCP:3, PCC:4)
Hunan Children’s Hospital	5(PCP:4, PCC:1)
Guangzhou women and Children’s medical center	5(PCP:4, PCC:1)
Underlying disease, n	
Primary immunodeficiency disease	24
Solid tumor	14
Hematologic malignant disease	4
HSCT	2
Solid organ transplantation	3
Rheumatic disease	2
Nephrotic syndrome	2
Inherited metabolic disease	2
Others	8
Fever, n(%)	52(88.1)
Mechanical ventilation, n(%)	51(86.4)
Co-infection, n(%)	46(78.0)
Cytomegalovirus, n(%)	21(35.6)
Epstein-Barr virus, n(%)	6(10.2)
Acinetobacter baumannii, n(%)	5(8.5)
Mycobacterium tuberculosis, n(%)	4(6.8)
Streptococcus pneumoniae, n(%)	4(6.8)
Anti-infection adjustment, n(%)	26(44.1)

*PCP* *Pneumocystis jirovecii* pneumonia, *PCC* *P. jirovecii* colonization, *HSCT* hematopoietic stem cell transplantation

### Comparison of clinical and molecular features between the PCP and PCC groups

**Fig. 2:**
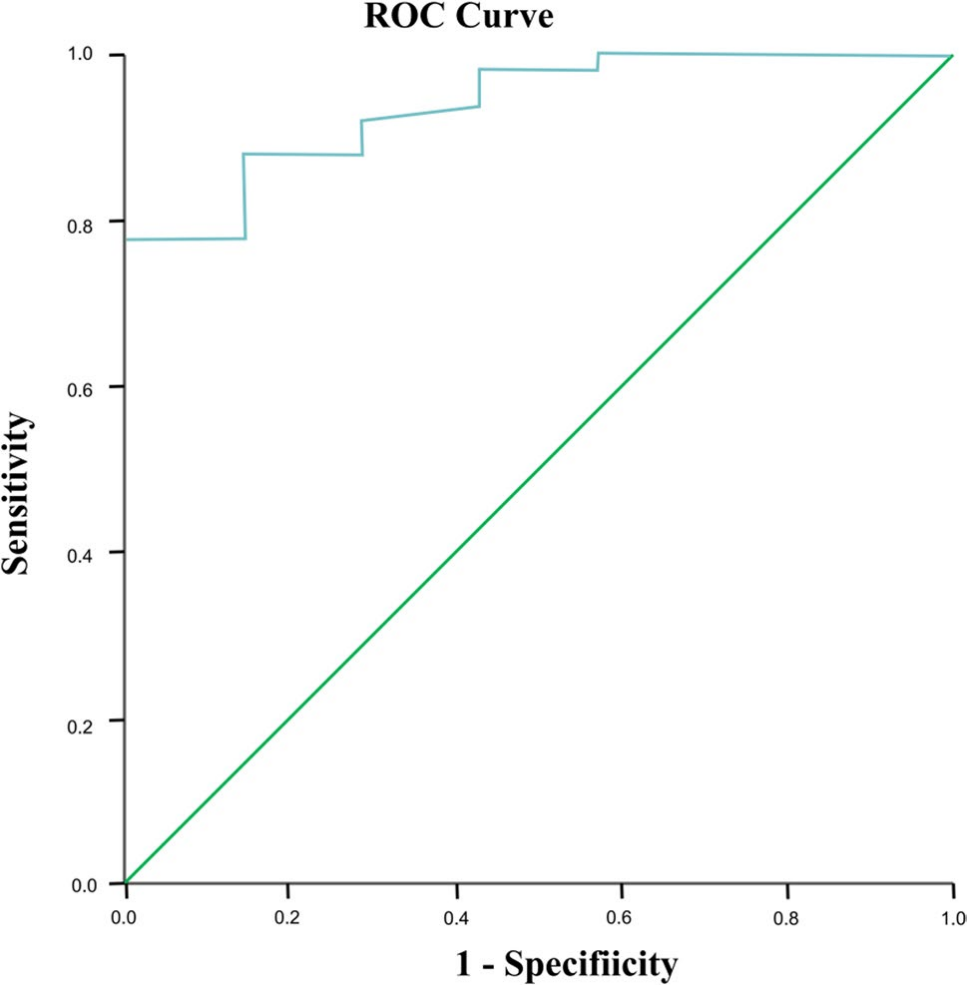
Receiver operating characteristic (ROC) curve of metagenomic next-generation sequencing analysis to distinguish *Pneumocystis jirovecii* pneumonia from *P. jirovecii* colonization

Serum C-reactive protein (CRP) levels were significantly higher in the PCP group than in the PCC group (*P* = 0.004). The median and IQR of serum lactate dehydrogenase (LDH) levels was higher in the PCP group (720 U/L; 539, 978) than in the PCC group (372 U/L; 279, 752), but the difference did not reach statistical significance. The median read number of *P. jirovecii* in mNGS analysis was significantly higher in the PCP group than in the PCC group (5,955 vs. 8, *P* < 0.001). The area under the curve for the *P. jirovecii* read number in mNGS of BALF was 0.937 (95% confidence interval: 0.865–1.000) (Fig. 2). The optimal threshold value for discriminating *P. jirovecii* infection from colonization appeared to be 556 reads (sensitivity, 77.6%; specificity, 100.0%), when the Youden index was at its maximum (0.776). There were no significant between-group differences in terms of age, weight, or sex (Table 2).

**Table 2:** Comparison of demographic and clinical characteristics between the PCP and PCC study groups

Variables	PCP	PCC	Z/t/X^2^	*P*
Number	*n* = 51	*n* = 8		
Age, months	10.00(5.00, 48.50)	7.50(2.50, 31.50)	1.164	0.244
Weight, kg	8.50(6.75, 14.00)	7.50(5.50, 11.50)	0.820	0.412
Male, n(%)	37(72.5)	5(62.5)	0.027	0.870
PELOD-2 zcore	4.32 ± 2.70	3.25 ± 3.95	0.736	0.465
Hosipital stay time, days	34.0(17.5, 48.5)	17.0(13.5, 23.0)	1.816	0.069
PICU stay time, days	21.0(10.5, 37.0)	13.0(7.5, 16.5)	1.600	0.110
Mechanical ventilation time, days	16.00(7.00, 27.00)	7.00(4.00, 11.50)	1.904	0.057
Fever, n(%)	46(90.2)	6(75.0)		0.238
WBC, 10^9^/L	6.10(4.03, 10.20)	8.81(5.72, 11.51)	1.218	0.223
CRP, mg/L	29.80(8.00, 83.50)	0.99(0.50, 14.93)	2.854	0.004
LDH, U/L	720(539, 978)	372(279, 752)	1.860	0.063
Reads number(DNA)	5955(595, 23971)	8(3, 69)	3.717	<0.001
Co-infection, n(%)	41(80.4)	5(62.5)	0.458	0.499
Mortality, n(%)	18(35.3)	2(25.0)	0.029	0.865

Data presented as mean ± standard deviation, median (P_25_, P_75_) or number (%). *PCP* *Pneumocystis jirovecii* pneumonia, *PCC* *P. jirovecii* colonization, *PELOD-2* Pediatric Logistic Organ Dysfunction 2, *PICU* pediatric intensive care unit, *WBC* white blood cell, *CRP* C-reactive protein, *LDH* lactic dehydrogenase, *BDG* 1,3-β-D-glucan, *HSCT* hematopoietic stem cell transplantation

### Prognostic comparison between survivors and nonsurvivors in the PCP group

To identify prognostic factors for PCP, the PCP group patients were subclassified into nonsurvivors (*n* = 18) and survivors (*n* = 33) based on discharge status. Compared with survivors, nonsurvivors were younger, had a lower body weight, and experienced shorter hospital stays (all *P* < 0.05). Median T-cell counts (IQR) were significantly lower in nonsurvivors than in survivors (all *P* < 0.05): CD3^+^, 126 (51, 590) vs. 542 (234, 1721) cells/µL; CD4^+^, 38 (4, 65) vs. 268 (64, 1015) cells/µL; and CD8^+^, 60 (6, 120) vs. 262 (121, 635) cells/µL. The CD4^+^/CD8^+^ ratio was also lower in nonsurvivors (14.55 [2.10, 32.57] vs. 27.91 [15.10, 45.53]; *P* < 0.05). The proportion of patients with primary immunodeficiency was significantly higher among nonsurvivors than survivors (*P* = 0.008), as was the PELOD-2 score at admission (*P* = 0.016) (Table 3). However, multivariable logistic analysis indicated that prognosis was not associated with age, weight, CD counts (CD3^+^, CD4^+^, CD8^+^, or CD4^+^/CD8^+^ ratio), PELOD-2 score at admission, or primary immunodeficiency. There was no significant difference in the median mNGS read count of *P. jirovecii* between subgroups.

**Table 3:** Comparison of survivor and nonsurvivor subgroups within the PCP cohort

Variables	Non-survivors	Survivors	Z/t/X^2^	*P*
Number	18	33		
Age, months	6.00(4.75, 21.50)	20.00(5.50, 67.00)	2.015	0.044
Weight, kilograms	7.50(5.88, 9.63)	11.00(7.00, 16.75)	2.061	0.039
Male, n(%))	12(66.7)	25(75.8)	0.135	0.714
PELOD-2 score	5.50 ± 2.64	3.59 ± 2.50	2.497	0.016
Hosipital stay time, days	20.5(9.5, 42.0)	38.0(22.5, 55.0)	2.277	0.023
PICU stay time, days	16.0(8.5, 34.0)	21.0(11.5, 40.5)	1.183	0.237
Mechanical ventilation time, days	13.00(6.00, 32.00)	19.00(8.00, 23.00)	0.418	0.676
Fever, n(%)	17(94.4)	29(87.9)	0.068	0.794
WBC, 10^9^/L	7.40(4.87, 10.30)	5.40(3.28, 11.00)	0.857	0.391
Lymphocyte, 10^9^/L	0.71(0.47, 1.81)	0.61(0.29, 3.07)	0.345	0.730
CRP, mg/L	33.27(8.32, 83.95)	28.00(5.32, 89.50)	0.850	0.395
LDH, U/L	814(612, 1340)	687(476, 916)	1.666	0.096
CD3, 10^6^/ml	126(51, 590)	542(234, 1721)	2.964	0.003
CD8, 10^6^/ml	60(6, 120)	262(121, 635)	3.460	0.001
CD4, 10^6^/ml	38(4, 65)	268(64, 1015)	3.137	0.002
CD4 ratio	14.55(2.10, 32.57)	27.91(15.10, 45.53)	2.350	0.019
Reads number(DNA)	4838(1285, 15329)	10,359(371, 40994)	0.294	0.769
BDG, pg/ml	185(151, 320)	455(171, 675)	2.250	0.024
Underlying disease, n(%)				
Primary immunodeficiency disease	13(72.2)	11(33.3)	7.070	0.008
Solid tumor	3(16.7)	11(33.3)	0.895	0.344
Solid organ transplantation	0(0.0)	2(6.1)		0.537
HSCT	1(5.6)	1(3.0)		1.000
Glucocorticoid, n(%)	13(72.2)	24(72.7)	0.000	1.000
Co-infection, n(%)	14(77.8)	27(81.8)	0.000	1.000
Ground-glass opacity	10(35.7)	25(40.3)	0.476	0.490

Data presented as mean ± standard deviation, median (P_25_, P_75_) or number (%). *PCP* *Pneumocystis jirovecii* pneumonia, *PELOD-2* Pediatric Logistic Organ Dysfunction 2, *PICU* pediatric intensive care unit, *WBC* white blood cell, *CRP* C-reactive protein, *LDH* lactic dehydrogenase, *BDG* 1,3-β-D-glucan, *HSCT* hematopoietic stem cell transplantation

### Comparison between primary and secondary immunodeficiency subgroups in the PCP cohort

The PCP group was stratified into primary immunodeficiency (*n* = 24) and secondary immunodeficiency (*n* = 27) subgroups based on underlying conditions, and intergroup differences were analyzed. Compared with the secondary immunodeficiency subgroup, patients with primary immunodeficiency had lower body weight, higher white blood cell and lymphocyte counts, and lower IL-6 expression (all *P* < 0.05). Median CD4^+^ T-cell counts (IQR) were 175 (12, 728) cells/µL in the primary subgroup and 77 (35, 524) cells/µL in the secondary subgroup, with median values below 200 cells/µL in both groups. The CD4^+^/CD8^+^ ratio was significantly higher in the primary immunodeficiency subgroup (*P* = 0.012), as was mortality at discharge (54.2% vs. 18.5%, *P* = 0.008). No significant between-subgroup differences were observed in hospital or PICU stay duration, rates of invasive/noninvasive mechanical ventilation, *P. jirovecii* read counts in BALF mNGS, or serum BDG levels (Table 4).

**Table 4:** Comparison of primary and secondary immunodeficiency subgroups within the PCP cohort

Variables	primary immunodeficiency	Secondary immunodeficiency	Z/t/X^2^	*P*
Number	24	27		
Age, months	6.00(4.00, 8.50)	41.00(15.50, 67.00)	3.659	0.000
Weight, kg	7.00(5.90, 8.15)	12.60(9.00, 16.75)	3.815	0.000
Male, n(%)	18(75.0)	19(70.4)	0.137	0.712
PELOD-2 score	4.55 ± 3.07	4.12 ± 2.37	0.536	0.595
Hosipital stay time, days	37.5(17.0, 48.0)	31.0(17.5, 51.0)	0.444	0.657
PICU stay time, days	21.0(11.5, 35.5 )	18.0(9.5, 39.0)	0.019	0.985
Mechanical ventilation time, days	19.0(9.0, 32.0)	14.0(6.0, 21.0)	1.445	0.148
Fever, n(%)	22(91.7)	24(88.9)	0.000	1.000
WBC, 10^9^/L	8.31(5.22, 12.20)	4.60(3.03, 8.22)	2.321	0.020
Lymphocyte, 10^9^/L	1.41(0.49, 2.80)	0.59(0.28, 1.13)	2.161	0.031
IL-6, ng/L	25.2(8.2, 113.6)	119.0(29.1, 276.2)	2.191	0.028
CD3, 10^6^/ml	579(51, 1398)	274(179, 1293)	0.227	0.821
CD8, 10^6^/ml	139(12, 551)	191(96, 516)	0.928	0.353
CD4, 10^6^/ml	175(12, 728)	77(35, 524)	0.144	0.885
CD4/CD8	1.70(1.00, 2.59)	0.77(0.31, 1.57)	2.516	0.012
Reads number(DNA)	8138(1642, 23591)	5431(590, 26679)	0.511	0.609
BDG, pg/ml	224(164, 521)	374(159, 619)	0.642	0.521
Mortality, n(%)	13(54.2)	5(18.5)	7.070	0.008

Data presented as mean ± standard deviation, median (P_25_, P_75_) or number (%). *PCP* *Pneumocystis jirovecii* pneumonia, *PELOD-2* Pediatric Logistic Organ Dysfunction 2, *PICU* pediatric intensive care unit, *WBC* white blood cell, *IL-6* interleukin-6, *BDG* 1,3-β-D-glucan

## Discussion

*P. jirovecii* is a life-threatening opportunistic pathogen and an important cause of severe pneumonia in immunocompromised children [19]. Among individuals without HIV infection, accurate and timely diagnosis is the most important factor for reducing PCP-related mortality. As a rapid, unbiased pathogen detection technology, mNGS offers high sensitivity, comprehensive coverage, and timely identification of infections in critically ill children [20–22]. Although many studies have demonstrated that mNGS exhibits high sensitivity for detecting *P. jirovecii* [7, 12, 13], distinguishing infection from colonization remains challenging. In this multicenter retrospective observational study, we focused on critically ill, HIV-negative children with *P. jirovecii*-positive BALF mNGS results, aiming to evaluate the diagnostic utility of mNGS in differentiating *P. jirovecii* infection from colonization and to delineate the clinical profile of pediatric PCP.

Our findings demonstrate the strong diagnostic performance of mNGS in distinguishing *P. jirovecii* infection from colonization, with an optimal threshold of 556 reads (sensitivity, 77.6%; specificity, 100.0%). In contrast, a study by Liu and colleagues reported a lower cut-off of 14 reads (sensitivity, 83.3%; specificity, 95.7%) and noted elevated levels of LDH and CRP in the PCP group compared with those in the PCC group [13]. These discrepancies likely reflect differences in patient populations and underlying conditions. In the absence of widely accepted mNGS thresholds, we should interpret mNGS results in conjunction with clinical characteristics and radiologic findings.

The unbiased and broad-spectrum nature of mNGS is poised to guide effective antimicrobial therapy. In the current study, 26 patients (44.1%) underwent antimicrobial regimen adjustments based on mNGS results combined with clinical and radiologic manifestations. This rate is slightly higher than that in our previous study, which reported a 36.1% modification rate [12]. Meanwhile, Jiang and colleagues observed a substantially higher adjustment rate of 71.7% based on mNGS results [7]. These studies underscore the potential of mNGS in guiding therapeutic decision-making in PCP.

Probable PCP is defined by compatible host factors, clinical and radiologic criteria, plus detection of *P. jirovecii* by quantitative PCR and/or positive serum BDG testing, provided false-positives and invasive fungal disease can be excluded [6]. However, PCR has limited diagnostic value for identifying coinfections, which are common in immunocompromised patients with PCP, while BDG is a highly sensitive but nonspecific serologic biomarker. As a rapid, unbiased diagnostic tool, mNGS enables detection of diverse pathogens in a single run [23]. Its utility in identifying PCP in HIV-negative patients has been well established, with high sensitivity and specificity reported across multiple studies [7, 12, 13]. In our current cohort, mNGS detected mixed infections in 78.0% of patients. The most common pathogen in the mixed infections was cytomegalovirus, consistent with previous related studies [7, 10, 11].

Solid tumors and hematologic malignancies were the most common underlying conditions reported in prior studies of PCP-infected adults without HIV infection [24, 25]. However, in our study, approximately 47.1% (24/51) of the children diagnosed with PCP were found to have a primary immunodeficiency. Differences in disease spectrum between adults and children may account for this discrepancy. Children with primary immunodeficiency were younger, had lower body weight, and experienced significantly higher mortality than those with secondary immunodeficiency. These findings align with our previous single-center retrospective study [12], supporting the notion that primary immunodeficiency may contribute to a poor prognosis in pediatric PCP. Appropriate prophylaxis should be considered in this high-risk population.

While the mortality rate of non-HIV-infected children with PCP in the current study was 35.3%, the reported mortality rates for this population ranged from 11.8% to 41.7% in prior studies [10, 11, 26]. Our analysis revealed that nonsurvivors were younger, with lower T-cell subset counts (CD3^+^, CD4^+^, and CD8^+^), a higher proportion of primary immunodeficiency disease, and higher PELOD-2 scores. However, these associations were not retained in multivariable logistic analysis. Ling et al. also reported younger age and a higher proportion of primary immunodeficiency among nonsurvivors of this population [26], with elevated LDH levels, coinfection, and need for mechanical ventilation as risk factors for a poor prognosis of PCP. Differences in the spectrum of underlying diseases in the two studies might contribute to this discrepancy. We also analyzed the mNGS *P. jirovecii* read counts between the survivor and nonsurvivor subgroups and found no statistical difference, consistent with a study by Wang et al. [27]. In their study, no significant difference was observed in *P. jirovecii* sequence counts between survivor and nonsurvivor groups, despite numerically higher values in the latter.

Our study has several limitations. First, the number of enrolled patients differed significantly between the PCC and PCP groups, which may have reduced the statistical power to detect true differences. Second, because PCR amplification of *P. jirovecii* was not performed at any of the four participating hospitals, a comparison between mNGS and PCR could not be conducted. Third, the turnaround time for completing all mNGS steps was 24–48 h, which exceeds that required for staining or PCR. Fourth, the study lacked a formal inter-rater reliability assessment. We will include this in the design of future prospective studies. Finally, data on prophylactic use of TMP/SMZ prior to BALF collection, which may have influenced mNGS read counts, were not available.

## Conclusion

The mNGS technology shows promise in distinguishing *P. jirovecii* infection from colonization, but more research is needed to validate its diagnostic thresholds and clinical utility.

## Supplementary Information

Supplementary Material 1.

## Data Availability

The datasets generated and/or analysed during the current study are available in the China National GeneBank Sequence Archive (CNSA), ( https://db.cngb.org/cnsa/), number CNP0007748.

## Abbreviations

PJ: *Pneumocystis jirovecii*
PCP: *Pneumocystis jirovecii* pneumonia
HIV: Human immunodeficiency virus
mNGS: Metagenomic next-generation sequencing
BALF: Bronchoalveolar lavage fluid
PCC: *Pneumocystis jirovecii *colonization
CRP: C-reactive protein
PICU: Pediatric intensive care unit
TMP/SMZ: Trimethoprim/sulfamethoxazole
BDG: 1,3-β-D-glucan
PELOD-2: Pediatric logistic organ dysfunction-2
ROC: Receiver operating characteristic curve
HSCT: Hematopoietic stem cell transplantation
LDH: Lactic dehydrogenase
