# Supplementary material for: Metagenomic next-generation sequencing to detect Pneumocystis jirovecii pneumonia in critically ill, HIV-negative children: a retrospective multicenter study
Source: BMC Pulm Med. 2026 Feb 7;26:114. doi: 10.1186/s12890-026-04163-9 (PMC12977887; doi:10.1186/s12890-026-04163-9)
Supplement: Supplementary file 1 — Supplementary Material 1. [file 12890_2026_4163_MOESM1_ESM.docx]

**Supplementary Table 1. Spectrum of co-detected pathogens on BALF mNGS: PCP vs. PCC Groups**

| **Co-infection pathogens, n(%)** | **PCP (n = 51)** | **PCC (n = 8)** |
| --- | --- | --- |
| Cytomegalovirus | 20(39.2) | 1(12.5) |
| Epstein-Barr virus | 6(11.8) | 0(0.0) |
| Torque teno virus | 3(5.9) | 0(0.0) |
| Human parainfluenza virus | 2(3.9) | 0(0.0) |
| Human respiratory syncytial virus | 0(0.0) | 1(12.5) |
| Other viruses | 4(7.8) | 0(0.0) |
| *Acinetobacter baumannii* | 5(9.8) | 0(0.0) |
| *Mycobacterium tuberculosis* | 4(7.8) | 0(0.0) |
| *Streptococcus pneumoniae* | 3(5.9) | 1(12.5) |
| *Elizabethkingia* spp. | 3(5.9) | 0(0.0) |
| *Burkholderia cepacia* complex | 3(5.9) | 0(0.0) |
| *Escherichia coli* | 2(3.9) | 0(0.0) |
| *Enterococcus faecalis* | 2(3.9) | 1(12.5) |
| *Rothia* spp. | 0(0.0) | 1(12.5) |
| *Mycoplasma pneumoniae* | 0(0.0) | 1(12.5) |
| *Haemophilus influenzae* | 0(0.0) | 1(12.5) |
| Other bacteria | 20(39.2) | 0(0.0) |
| *Candida* spp. | 3(5.9) | 0(0.0) |
| Other fungi | 1(2.0) |  |

Data presented as number (%). BALF: Bronchoalveolar lavage fluid; mNGS: Metagenomic next-generation sequencing; PCP: *Pneumocystis jirovecii* pneumonia; PCC: *P. jirovecii* colonization.
